# Cognitive Development of Children with Craniosynostosis

**DOI:** 10.15844/pedneurbriefs-29-6-6

**Published:** 2015-06

**Authors:** J. Gordon Millichap

**Affiliations:** 1 Division of Neurology, Ann & Robert H. Lurie Children's Hospital of Chicago, Chicago, IL; 2 Departments of Pediatrics and Neurology, Northwestern University Feinberg School of Medicine, Chicago, IL

**Keywords:** Craniosynostosis, Intelligence, Learning

## Abstract

Investigators from University of Washington, Seattle, WA; Harvard U, MA; St Louis, MO; Atlanta, GA; Northwestern U, and Shriner's Hospital, Chicago, compared the development of school-aged children with single-suture craniosynostosis (sagittal, metopic, unicoronal, lambdoid) and unaffected children.

Investigators from University of Washington, Seattle, WA; Harvard U, MA; St Louis, MO; Atlanta, GA; Northwestern U, and Shriner's Hospital, Chicago, compared the development of school-aged children with single-suture craniosynostosis (sagittal, metopic, unicoronal, lambdoid) and unaffected children. Tests of intelligence, reading, spelling, and math were administered in 182 case participants and 183 controls. Case participants’ average scores were lower than controls on all measures. Full-Scale IQ and math computation showed the largest differences; case participants’ adjusted mean scores were 2.5 to 4 points lower than those of control participants (Ps ranged from .002 to .09). Mean case-control differences on other measures of achievement (reading and spelling), partially evident at school age, were only slightly lower, but case deficits were more pronounced after adjusting for participation in developmental interventions. The frequency of specific learning problems in case and control participants was comparable; among case participants, 58% had no learning problem. Children with metopic, unicoronal and lambdoid synostosis tended to score lower on most measures than those with sagittal fusion (P<.001 to .82). In patients with single-suture fusions, neurodevelopmental screening in preschool years is especially important in those with unicoronal and lambdoid synostosis, with more selective screening of children with isolated sagittal fusions. [1]

COMMENTARY. This 10-year, multi-site study of the cognitive development of children with single-suture craniosynostosis shows that children born with the disorder are on average more likely to develop learning problems in early elementary school. Developmental delays are generally mild and vary significantly, those with unicoronal or lambdoid synostosis being most vulnerable, whereas sagittal synostosis cases, the most common variety of synostosis, are spared. Boys with single-suture craniosynostosis score lower on academic and IQ tests than girls; and males are more likely than females to have learning problems (50 vs 30%); males with unicoronal synostosis have a 86% risk of learning disorder [2].

The cause of neurodevelopmental and cognitive delay of infants with single-suture craniosynostosis remains unclear [3]. Craniosynostosis is frequently complicated by other neurological abnormalities constituting various syndromes, eg Apert syndrome (acrocephalopolysyndactyly), sometimes associated with cerebral malformation and hydrocephalus [4]. Various cognitive profiles are described in patients with Apert syndrome [5]. Other syndromes that list craniosynostosis as a major abnormality include Crouzon, Pfeiffer, Carpenter, Jackson-Weiss, Saethre-Chotzen, Beare-Stevenson, Vogt, Waardenburg, and Muenke syndrome. The characteristics of Muenke syndrome are a unilateral coronal craniosynostosis with anterior plagiocephaly, asymmetry of skull and face, developmental delay and learning disorder. This unilateral craniosynostosis is explained by a mutation in the gene FGFR3 [6].
